# Sialoglyco-Conjugate Abnormalities, IL-6 Trans-Signaling and Anti-Ganglioside Immune Response—Potential Interferences in Lupus Nephritis Pathogenesis

**DOI:** 10.3390/diagnostics11061129

**Published:** 2021-06-21

**Authors:** Corina-Daniela Ene, Mircea Nicolae Penescu, Ilinca Nicolae

**Affiliations:** 1 Internal Medicine and Nephrology Department, Carol Davila University of Medicine and Pharmacy, Eroilor Sanitari Street, No 8, 050474 Bucharest, Romania; mirceapenescu4@gmail.com; 2 Nephrology Department, Carol Davila Clinical Hospital of Nephrology, Grivitei Street, No 4, 010731 Bucharest, Romania; 3 Research in Dermatology Department, Victor Babes Clinical Hospital of Tropical and Infectious Diseases, Mihai Bravu Street, No 281, 030303 Bucharest, Romania; drnicolaei@yahoo.com

**Keywords:** sialoglyco-conjugates, IL-6 signaling pathways, anti-ganglioside antibodies, systemic lupus erythematous, lupus nephritis

## Abstract

We have investigated glycoconjugates sialization profile, endogen synthesis rate of antiganglioside antibodies (AGA), IL-6 signaling pathways correlated with activity disease in systemic lupus erythematous (SLE) and lupus nephritis (LN). Material and methods. A case-control study was developed and included 109 patients with SLE with or without renal impairment, 32 patients with IgA nephropathy and 60 healthy volunteers, clinically and paraclinically monitored. The following parameters were evaluated in volunteers serum: total sialic acid (TSA), orosomucoids, lipid bound sialic acid (LSA), interleukin-6 (IL-6), soluble factors IL-6R, gp130, anti –GM1, -GM2, -GM3, -GD1a, -GD1b, -GT1b, -GQ1b antigangliosides antibodies of IgG and IgM type. Results. Experimental data analysis showed: increase in synthesis rhythm of sialoglyco-conjugated in SLE (TSA increased in SLE and LN compared to control), accelerated catabolism of LSA in LN (LSA/TSA ratio was higher in SLE and LN than in control group), overexpression of IL-6 mediated trans-signaling (sIL-6R/sgp 130 ratio was subunit in SLE and IgA nephropathy and superunit in LN), large AGA profile synthesis of IgM isotype (over 45.1% in SLE and over 20.7% in LN). Conclusions. Hypersialization, accelerated glycosphingolipids degradation, IL-6 trans-signaling amplify and AGA pattern could represent essential mechanisms in LN pathogenesis.

## 1. Introduction

N-acethyl-neuraminic acid (acid sialic, Neu5Ac, NANA) is a negatively charged molecule, found as terminal monozaharide in glyco-conjugates, essential metabolite in human physiology and pathology. Sialic acid, in the active form of CMP-Sia, is transported from cellular nucleus in Golgi apparatus. Sialic acid residues, under ST3, ST6, ST8 syalyl-transferases action are transferred on glyco-proteins and glycol-lipids [1,2,3,4,5,6,7]. In human physiology, syalyl-conjugates are involved in cells interaction, cellular aggregation, intercell communication and signaling, development of immunity and renal function. Aberrant expression of carbohydrates on cells surface are associated with malignant transformation, microbiome status, viral infections, altered immune response [1,7,8,9,10]. Circulant glyco-proteins (orosomucoids or alpha-1-acid glycoprotein) have important functions in immune and inflammatory response and could play a primary role in SLE disease activity and relapse [11,12]. Glycosphingolipids are involved in autoimmune diseases pathogenesis and renal disfunctions [13,14,15]. These molecules were little investigated in lupus nephritis (LN) during years [10].

Glicosphingolipids modulate many events in SLE and podocytopathies [16,17]. These biomolecules are involved in cell lipid rafts organization, alteration of signal transduction pathways, ligands-cell receptor interaction. Neutral sphingolipids that include sphingomyelin, ceramides, Glc-Cer, Lac-Cer, globe-trihexaosil-ceramide, ganglio-tetraosil-ceramide, globo-pentaosil-ceramid and anionic sphingolipids that include GM3, GM1b, GD1 were detected in human podocytes [16]. O-acethyl-GD3 was highly expressed in rat kidney with lead, Adriamycin or puromycin induced glomerulopathy. O-Acethyl-GD3 overexpression was correlated with decreased GD3, inactivation of GD3-synthesis and O-acetyltransferase. GD3-O-acetilain has been linked to viral infections, resistance to apoptosis, proteinuria, regulation of podocytes function [16]. GM3 assures interaction between vascular endothelial growth factor (VEGF), endothelial growth factor (EGF), insulin and their receptors on podocytes surface, cytoskeleton reorganization, filtration barrier integrity, glomerular permeability [16,17,18]. In LN patients, some studies showed increased hexozilceramides and LacCer, ST3 and NEU alteration [19]. These data suggest a disequilibrium between gangliosides synthesis and their catabolism in LN pathogenesis.

Gangliosides are involved in myeloid, lymphoid cells and stem cells function and an alteration in gangliosides metabolism could be involved in immune pathogenesis of SLE. GM3 could be found mostly in immune cells, while GM1 and asialo-GM1 in eosinophiles, basophiles and natural killers (NK) cells. O-acetyl-GD3 was found in T, B and NK cells. High expression of GM3-sinthase in kidney concomitant with high Bcl-2 suggest the protective role of GM3 in kidney by anti-apoptosis mechanism [16]. Monosialo-ganglioside GM1 is expressed in CD4 positive T cells in SLE patients and regulates LCK activity by CD 45 tyrosine-phospho-kinase [17].

Glicosphingolipids alteration in kidney was associated with high activity of neuraminidase (sialidase), which modulates mesangial cells capacity of IL-6 synthesis. IL-6 family has ten representants: IL-6, IL-11, IL-27, IL-31, leukemia inhibitory factor (LIF), M oncostatin (OSM), cardiotrohpin-1 (CT-1), ciliary neurotrophic factor (CNTF), neuropoietin (NP), cardiotrophin-like cytokine (CLC). These cytokines actions are modulating mechanisms of human organism immune and inflammatory response. IL-6 is secreted by macrophages, lymphocytes, fibroblasts, synoviocytes, keratinocytes, chondrocytes, renal-resident cells, including podocytes, mesangial cells, endothelial cells, and tubular epithelial cells [10,20]. The specific IL-6 receptor is a protein membrane complex, composed by a structural subunit IL-6R alfa and a signaling subunit gp130, common component of many cytokine receptors. IL-6R is expressed on macrophages, neutrophiles, CD4 positive T cells, podocytes, hepatocytes. These cells respond to IL-6 and activates membranous gp130 through membrane alpha IL-6R and initiates classical signaling, associated with anti-inflammatory effects. IL-6 has also, proinflammatory effects (trans-signaling) by IL-6-soluble receptor (sIL-6R)—gp130 (a membrane glycoprotein expressed on many somatic cells) interaction [21,22,23]. The experimental data showed that gp130/IL-6R alfa ratio induce stimulation of trans/classic IL-6 mediated signaling, via intracellular JAK/STAT3 and SPH2/Gab/MAPK pathways signaling [24]. If gp130 expression is higher than IL-6Ralfa, trans-signaling is more active than classical signaling. If IL-6Ralfa expression is higher than gp130 expression, both signaling pathways are overexpressed [21,22,23,24]. IL-6 is overexpressed in kidney, and through its proinflammatory actions, it plays a crucial role in membrane-proliferative glomerulonephritis, IgA nephritis, lupus nephritis, diabetic nephropathy, acute kidney injury, chronic kidney disease [25,26,27,28].

Altered expression of sphingolipids is recognized by specific antiganglioside antibodies (AGA). Endogen synthesis of AGA was observed in neoplasia [5,6,29,30,31], viral infections [32,33], in autoimmune diseases like multiple sclerosis, rheumatoid arthritis, Sjogren syndrome, diabetes [17,34,35,36,37,38]. AGA were studied in SLE patients, mostly on neuro-psychiatric and peripheric neuropathies, but without concluding results [39,40,41,42,43,44].

The present study aimed to analyzed the sialylation profile of glyco-proteins and glyco-sphingolipids, the potential endogen immune reactivity of modified gangliosides, IL-6 signaling and the analyses of the changes in relation to clinical data in SLE.

## 2. Materials and Methods

### 2.1. Study Participants

The present study is a case control study developed for a period of three years (2018–2020) including 109 SLE patients, 32 patients with IgA nephropathy and 60 healthy subjects. All the patients signed the informed consent, and all the procedures were performed according to the Declaration of Helsinki from 1975. Patients were selected from those who attended the Clinical Hospital of Nephrology “Carol Davila” and Clinical Hospital “Victor Babes”, and the study protocol was approved by the Ethics Committee of Clinical Hospital of Nephrology “Carol Davila” (13–27 July 2018). Of these 109 SLE patients, 62 had SLE with cutaneous and hematological determinations, but no lupus nephritis (non-LN group), while 47 had lupus nephritis (LN group). The patients with lupus nephritis or IgA nephropathy were diagnosed by biopsy puncture and histological exam according to KDIGO guidelines. In LN patients, 8% had type II LN, 16% had type III LN, 72% had type IV and 4% type V. SLE diagnosis was established according to Systemic Lupus International Collaborating Clinics/American College of Rheumatology criteria. The activity disease was based on clinical Systemic Lupus Erythematosus Disease Activity Index (SLEDAI). The time lapse of disease, ongoing treatment (non-steroidal anti-inflammatories, immunosuppressant drugs like corticosteroids, hydroxychloroquine, azathioprine, mycophenolate mophetil, antihypertensive therapy) were recorded for each patient. In the control group, we enrolled healthy subjects over 18-years-old, with adequate nutritional status. The exclusion criteria were: cardiovascular, hepatic, thyroid, gastrointestinal, oncological disease, recent history of viral or bacterial infections, tobacco use, drug abuse, alcoholism, use of vitamin or other antioxidant supplements, pregnancy. Patients’ characteristics are presented in Table 1.

### 2.2. Laboratory Data

The blood samples were collected from all the study participants, after 12 h of fasting, using a holder-vacutainer system. Centrifugation of the blood samples was made at 3000× *g*, for ten minutes, after one hour of keeping the blood at room temperature. The sera were separated and frozen at −80 degrees before analyzing. We excluded the hemolyzed, icteric, lactescent, or microbiologically contaminated samples. The samples for laboratory determinations were collected from after signing the informed consent.

Sialic acid was dosed using resorcinol-chlorohydric acid. Blue chromophore was extracted with n-butyl/n-butanol acetate and spectrophotometry measured at 580 nm with the Sigma reactive (SIALICQ kit) and the BS3000 analyzer (SINNOWA Medical Science and Technology, Nanjing, China). Circulant levels of orosomucoids were measured by immunonephelometry at 340 nm with Human reactives (MBS901995 kit) in the HumanStar300 analyzer (HUMAN Gesellschaft für Biochemica und Diagnostica mbH, Weisbaden, Germany).

sIL-6R and sgp130 were assessed by the sandwich ELISA method, using immunoenzymatic kits (R&D SYSTEMS (DR600 and SRG00 kits), Minneapolis, MN, USA), the results being evaluated at 450 nm, using a TECAN analyzer (Tecan, Männedorf, Switzerland).

The assessment of anti-ganglioside antibodies was made by immunoblot technique, using EUROLine kits. This method allows the in vitro evaluation of antibodies, IgG and IgM classes, against GM1, GM2, GM3, GD1a, GD1b, GT1b, GQ1b from serum/plasma. The kit contains strips marked with purified antigens. The evaluation of antiganglioside antibodies was performed using the EUROLine Scan software. After reading the signal intensity on the strips marked with ganglioside antigens, the results were evaluated and the results are presented as optical sensibility.

### 2.3. Statistical Analysis

The data were presented using the mean and standard deviation. The comparison of data between groups were undertaken using either ANOVA with Tukey post hoc test or Kruskal–Wallis test with Dunn’s post hoc test for normally and non-normally distributed data, respectively. The relation between the studied markers was assessed by Pearson’s correlation coefficient, not before the assessment of data normality by the Kolmogorov–Smirnov test. The level of significance (*p*) chosen was 0.05 (5%) and the confidence interval was 95% for hypothesis testing and the corresponding ethical approval code

## 3. Results

### 3.1. Clinical Characteristics of the Studied Groups

Clinical characteristics of the studied groups are presented in Table 1. Leucocytes and hemoglobin were statistically significant lower in SLE and LN groups compared with the control group (*p* ˂ 0.05), but without statistical variation between LN and SLE subjects (*p* > 0.05). Albumin was statistically significant lower in SLE and LN groups, when compared with control (*p* ˂ 0.05) and also in LN compared with SLE group (*p* ˂ 0.05). Serum creatinine was found to be lower in LN group than in SLE group (*p* ˂ 0.05) or in control group (*p* ˂ 0.05). Inflammation was assessed by C reactive protein, and we found high inflammation in SLE and LN groups compared with control (*p* ˂ 0.05) and in LN compared with SLE group (*p* ˂ 0.05). In IgA nephropathy group, clinical characteristics of the patients were similar with those in control group, except serum creatinine and hematuria (*p* ˂ 0.05).

### 3.2. Activity Disease—Clinical and Paraclinical Data

Classic biomarkers of lupus activity like SLEDAI, anti-ds DNA, UACR, C1q, C3 and C4 complement proteins were assessed and presented in Table 2. SLEDAI varied significantly between SLE and LN groups (*p* ˂ 0.05). dsDNA was statistically significant higher in LN and SLE groups when compared with control group (*p* ˂ 0.05), but it did not vary between SLE and LN groups (*p* > 0.05). Renal tubular injury was evaluated by measuring the urinary levels of b2-microglobulin, that was found to be higher in LN group than in SLE group (*p* ˂ 0.05) or in control group (*p* ˂ 0.05). Urinary albumin: creatinine ratio was statistically significant higher in SLE and LN groups compared with control (*p* ˂ 0.05), but it did not vary significantly between these groups (*p* > 0.05). C1q, C3 and C4 complement proteins were statistically significant higher in LN group than in SLE one (*p* ˂ 0.05) or in control group (*p* ˂ 0.05).

In the IgA nephropathy group, urinary b2-microglobulin and UACR had statistically significant higher levels compared to the control group (*p* ˂ 0.05).

### 3.3. Sialoglyco-Conjugates Metabolism Reprogramming

We evaluated sialylation status in SLE and control groups by determining serum levels of total sialic acid (TSA), lipid bound sialic acid (LSA), orosomucoids. Results are presented in Table 3. TSA increased 2.94 fold in SLE group (*p* < 0.01), respectively 4.88 folds in LN group (*p* < 0.01) when compared with control group. It increased in LN group 1.65 folds when compared with SLE groups (*p* < 0.01) and 3.81 folds when compared with IgA nephropathy group (*p* < 0.05). Orosomucoids increased 9.54 folds in SLE group (*p* < 0.01), respectively 10.37 folds in LN group (*p* < 0.01), when compared with control group, but it had no statistical significant variation between SLE groups (*p* > 0.05). Orosomucoids increased 4.54 folds in LN group when compared with IgA nephropathy group (*p* < 0.05). LSA increased 5.29 folds in SLE group (*p* < 0.01), respectively 4.01 folds in LN group (*p* < 0.01) when compared with control group, and 1.30 folds in SLE group compared with LN group (*p* < 0.05). LSA/TSA ratio was 1.80-folds higher in SLE group (*p* < 0.01) and 1.2-folds lower in LN group (*p* < 0.05), when compared with control group and 2.16-folds higher in SLE when compared with LN group (*p* < 0.01). In IgA nephropathy group, TSA was 1.36-folds higher (*p* = 0.05), while orosomucoids was 2.28 folds (*p* < 0.05), compared with control group. LSA and LSA/TSA ratio had no significant variations when compared with other groups (*p* > 0.05).

### 3.4. IL-6 Signaling Pathways Alterations

IL-6 signaling was evaluated by assessing serum IL-6, sgp 130 and sIL-6R/sgp 130 ratio. Results are presented in Table 4. IL-6 increased 5.40 folds in SLE group (*p* < 0.01), respectively 5.78 folds in LN group (*p* < 0.01), when compared with control group and 1.58 folds when compared with IgA nephropathy group (*p* < 0.05). This marker did not vary significantly between SLE groups (*p* > 0.05). sIL-6R increased 2.23 folds in SLE group (*p* < 0.01), respectively 2.48 folds in LN group (*p* < 0.01), when compared with control group. sIL-6R was 1.11 higher in LN group compared with SLE one (*p* < 0.05) and 1.19 folds when compared with IgA nephropathy group (*p* < 0.05). sgp 130 decreased 1.28 folds in SLE group (*p* < 0.01), respectively 1.59 folds in LN group (*p* < 0.01), when compared with control group. sgp 130 decreased 1.23 folds in LN compared with SLE group (*p* < 0.05) and 1.9 folds when compared with IgA nephropathy group (*p* <0.05). sIL-6R/sgp 130 ratio was 2.93-folds higher in SLE group (*p* < 0.01), respectively 4.06 folds in LN group (*p* < 0.01), when compared with control group, respectively 1.38 folds in LN compared with SLE group (*p* < 0.05) and 1.43 folds when compared with IgA nephropathy group (*p* < 0.05).

### 3.5. Immune Response Against Gangliosides

Immune response against gangliosides was assessed by anti- GM1, GM2, GM3, GD1a, GD1b, GT1b, GQ1b antibodies, of IgM and IgG type. Results are presented in Table 5. In SLE group, we detected high signals for IgM (%) and IgG (%) anti-GM1 in 45.1, respectively, 20.7, anti-GM2 in 28.0, respectively, 10.9, anti-GM3 in 15.8, respectively, 7.3, anti-GD1a in 13.4, respectively, 4.8, anti-GD1b in 8.53, respectively, 1.22, anti-GT1b in 9.75, respectively, 0.0, anti-GQ1b in 12.1, respectively, 0.0; moderate signal for IgM (%) and IgG (%) anti -GM1 in 31.7, respectively, 17.7, anti-GM2 in 8.53, respectively, 4.87, anti-GM3 in 9.75, respectively, 6.09, anti-GD1a in 4.87, respectively, 0.0, anti-GD1b in 3.65, respectively, 0.0, anti-GT1b in 7.3, respectively, 0.0, anti-GQ1b in 6.09, respectively, 0.0. In LN group, we detected high signals for IgM (%) anti -GM1 in 9.75, anti-GM2 in 2.44, anti-GM3 in 6.81, anti-GT1b in 11.3, anti-GQ1b in 4.54; moderate signal of anti -GM1 in 15.9, anti-GM2 in 15.9, anti-GM3 in 6.81, anti-GD1b in 11.3, anti-GT1b in 22.7. In IgA nephropathy group, AGA were not detectable. In control group, AGA were not detectable. Anti-GM1, -GM2,-GM3 IgM and IgG type varied significantly when compared SLE groups together, respectively with control group and IgA nephropathy group. Anti-GD1a, GD1b, GT1b, GQ1b antibodies IgM type varied significantly between groups, while IgG type had no significant variation between groups.

### 3.6. Interplay between Sialoglyco-Conjugates Abnormalities and Inflammatory Response

The results of assessment of the relation between sialoglyco-conjugates abnormalities and inflammatory response are presented in Table 6. In SLE group, we detected a high positive correlation between TSA-IL6, CRP, orosomucoids—IL-6, sIL-6R and CRP, LSA-CRP. When evaluated AGA, we observed positive correlation Ig M-anti-GM1-IL-6, CRP, IgM-anti-GD1a-CRP and negative correlation IgM-anti-GM2-CRP, IgM -anti-GM3-CRP. In LN group, we detected a positive correlation between TSA-IL6, CRP, orosomucoids–IL-6, sIL-6R and CRP, and negative correlation between LSA-IL-6, sIL-6R/sgp130. When evaluated AGA, we observed positive correlation Ig M-anti-GM1-IL-6, sIL-6R, CRP, IgM-anti-GD1a-CRP. In LN group, we detected a strong positive correlation between sIL-6R-IL-6, CRP.

### 3.7. Interplay between Sialoglyco-Conjugates Abnormalities and Disease Activity

The results of the relation between glycoconjugates/antigangliosides and disease activity markers assessment are presented in Table 7. In SLE group, TSA correlated statistically significant positive with SLEDAI, anti-DNA ds, UACR, urinary β2-microglobulin and negative with C3 and C1q. Orosomucoids correlated statistically significant positive with SLEDAI, anti-DNA ds, UACR. LSA correlated statistically significant positive with SLEDAI, anti-DNA ds, UACR and negative with C1q. AGA analysis showed IgM-anti-GM1 positive correlation with SLEDAI, anti-DNA ds, respectively negative with C3, and IgM-anti-GM2, IgM-anti-GM3 positive correlation with anti-DNA ds. In LN group, TSA correlated statistically significant positive with SLEDAI, anti-DNA ds, UACR, urinary β2-microglobulin and negative with C3 and C1q. Orosomucoids correlated statistically significant positive with SLEDAI, anti-DNA ds, UACR. LSA correlated statistically significant positive with SLEDAI, UACR. AGA analysis showed IgM-anti-GM1 and IgM-anti-GM2 positive correlation with anti-DNA ds.

The assessment of the relations between glycoconjugates/antigangliosides and disease activity markers in IgA nephropathy group showed a positive correlation between IL-6 and TSA (*r* = 0.39, *p* = 0.028), respectively orosomucoids (*r* = 0.28, *p* = 0.041).

## 4. Discussion

Modified metabolism of glycoconjugates with sialic acid has been investigated in this study in SLE and LN patients. It should be noted that, TSA, orosomucoids and circulating LSA were significantly increased when compared SLE and LN groups, respectively with IgA nephropathy and control groups. Increased serum levels of TSA and orosomucoids had a positive association with inflammatory factors: CRP, IL-6, sIL-6R markers of acute phase response patients, though they can be considered markers of acute phase response in SLE and LN. The alteration of circulating LSA had a weak correlation with inflammatory process. The precise mechanisms by which the metabolism of glycoconjugates affects the physiology of SLE and LN have not been elucidated. In our study, the level of the LSA/TSA ratio had higher values in SLE compared to control, and significantly lower values in LN compared to control and LSA correlated with IL-6, respectively, sIL-6R/sgp130, which denotes a link between glycosphingolipids catabolism and IL-6 trans-signaling in SLE. Based on our results, the LSA/TSA ratio could represent a potential molecular target in the early LN.

Studies in literature showed that sialoglyco-conjugates are abundant in kidneys, and they could determine SLE organ determinations [11,12,13,14,15]. Seromucoids are released in the early stages of inflammation, act as acute phase reactants, modulate immunity, maintain the barrier function of the capillaries by mediating sphingolipids metabolism [12]. Some researchers consider plasma and urinary glycosphingolipids useful tools in the early identification of LN [13,14,15]. Circulating sphingolipids (ceramides, hexosil-ceramides) were increased, and sphingoid bases were decreased in SLE patients versus control. Ceramide C16:0/sfingozin-1-phosphate ratio was associated with LN activity. Hexosil-ceramides C16:0 and C24:1 made the difference between active and inactive SLE [14]. Concentrations of specific ceramides (C18:0, C20;0, C24;1) were increased in plasma, serum and biopsies in patients with LN and altered renal function, compared to SLE without renal determinations and control [15]. Some studies mentioned high circulating sialic acid levels in SLE and rheumatoid arthritis, without reference to glomerular function [10]. In the present study, we obtained significant positive relation between sialic acid pattern and SLEDAI, anti-ADS, UACR and negative one with C3, C1q.

Abnormal responses of T cells in SLE were associated with an abnormal ganglioside profile [16]. Being polyanionic components of glomerular glycocalyx, gangliosides could influence the selectivity of glomerular filtering, and, though, a relation between microalbuminuria, eGFR reduction and glomerular hyposialilation in LN [6,10] could be considered. Neuraminidasic activity (NEU), involved in sialoglyco-conjugate catabolism, plays an important role in the response of mesangial cells predisposed to LN. By direct desialilation of glycoproteins and glycolipids, NEU could regulate the production of IL-6 [27,28]. Further studies intend to identify mechanisms of LN progression, mediators and molecular mechanisms by which glycosphingolipid catabolic pathway is overexpressed in lupus kidneys [27,28]. NEU1 and NEU3 expressions overlap with the binding of IgG to the cellular surface of mesangial cells predispose to lupus attack. These data suggest that NEU activity could induce nephritis in mesangial cells by a complex signaling pathway of IgG receptors [27].

Alteration of glycosphingolipids has been associated with disruption of secretion and signaling of IL-6 in patients with SLE and LN. The renal cells, immune cells and inflammatory cells have the ability to produce and secrete IL-6 by TLR4-p38/ERK-MAPK signaling pathway [25,45]. It is currently known that IL-6 titer could be used in the diagnosis of immune/inflammatory diseases and in treatment monitoring, in correlation with clinical and paraclinical data. Recent data suggest that IL-6 should be considered not only a mediator of inflammation, but also a disease marker [25,26]. IL-6 has been studied as disease activity marker in IgA nephropathy, being associated since very long with proteinuria worsening [46]. The present study evaluated IL-6 profile in IgA nephropathy and assessed higher levels of IL-6, sIL-6R and sIL-6R/sgp 130 compared with control group, but significant lower than in LN, respectively lower sgp 130 than in control group, but higher than in LN. IL-6 positive correlated with TSA and orosomucoids. In IgA nephropathy, the mesangial deposited molecular polymeric IgA1 and complement component can promote secretion of IL-6, and though its serum and urine levels are elevated and correlated with disease evolution [25]. Aberrant glycosylation of IgA is considered central in the pathogenesis of IgAN. Some studies showed that IL-6 could induce overproduction of aberrantly glycosylated IgA in a murine model of IgAN by APRIL production via TLR9 activation pathway, although exacerbating renal injury [47,48].

IL-6 is upregulated in the kidneys and was crucial in the onset of nephritis in lupus mice [27,28]. In patients and mice with SLE, IL-6 was shown to regulate DNAds levels, CD5 expression by DNA methylation, and activation of autoreactive B cells [49]. In LN, IL-6 level correlated with disease activity, proteinuria, hematuria, macrophage expansion, TCD4 +, TCD8 + lymphocyte infiltration, IgG and C3 deposition and fixation in the kidneys [24,48,50]. IL-6 was proved to be involved in tubulointerstitial fibrosis, tubular atrophy, acute and chronic lesions in immune renal diseases, like LN, in metabolic, ischemic, toxemic diseases [25,51].

Our study evaluated the relation between sialoglyco-conjugate abnormalities, gangliosides and IL-6 in SLE and LN, and also, glycosphingolipids alteration influence on IL-6 signaling in these autoimmune disease. Our results showed a negative relationship between the amount of LSA and IL-6 level, between LSA and the sIL-6R/sgp130 ratio and no relation between IL-6 and AGA in LN patients. IL-6 is known to promote the expression of sialyltransferases ST3GAL6, ST6GAL2 and sulfotransferases CHST4, CHST6, a phenomenon associated with hypersialilation of glycosphingolipid epitopes [49]. It is a debate about IL-6 signaling pathway in various human diseases and animal models, but the results are discordant. Recently, the concept that the ratio between the functional subunit gp130 and the structural subunit IL-6R alpha in cell membranes define the type of IL-6 signaling was developed [21]. The ratio sIL-6R to sgp130 could provide information on the modulatory mechanisms of IL-6 in the inflammatory response. In the present study, IL-6 was overexpressed in SLE and LN compared to control. The ratio sIL-6R/sgp130 was subunitary in SLE and IgA nephropathy and supraunitary in LN. In other words, the amount of sIL-6R was lower compared to sgp130 in SLE and IgA patients, though we could consider the simultaneous activation of the two IL-6-mediated signaling pathways in SLE and IgA nephropathy. In contrast, the amount of sIL-6R is higher compared to sgp130 in patients with LN, though it could be associated with overexpression of IL-6-direct trans-signaling. Specifically, the sIL-6R/sgp130 ratio could be a buffer that, in excess of sIL-6R, could promote trans-signaling, and, in excess of sgp130, could attenuate/block trans-signaling. Initially, sgp130 was described as a specific natural inhibitor of trans-signaling, as an IL-6-induced signaling antagonist [22]. sgp130 has long been thought to competitively inhibit trans-signaling without affecting classical signaling and therefore could be used as a molecular tool to differentiate trans-signaling from classical IL-6-mediated signaling. It has then been reported that, at elevated concentrations, sgp130 blocks even classical signaling by sequestering the free cytokine IL-6 in IL-6/sIL-6R/sgp130 complexes [49]. Our results sustain the role of the sIL-6R/sgp130 ratio in differentiating patients with SLE and LN by controlling classical and trans- IL-6 mediated signaling. Consequently, blocking IL-6 trans-signaling could attenuate the inflammatory response in patients with SLE. Currently, it is estimated that sgp130, a specific inhibitor of IL-6 trans-signaling, could be useful in limiting renal inflammation.

The probability that some gangliosides generate an immune response in SLE patients with cutaneous or renal determinations, their relation with sialic acid circulating pattern, IL-6-mediated signaling and correlation with disease activity were the goal of this multicenter unique study in medical literature. The analysis of anti-GM1, -GM2, -GM3, -GD1a, -GD1b, -GT1b, -GQ1b, IgG and IgM antibodies in patients with SLE, LN and control group, showed an interdependence between the modified sialylation of gangliosides and AGA synthesis rate in patients versus control. Our results showed differences in AGA-IgM signal strength in SLE and LN patients. The differences in AGA-IgG between groups were insignificantly. Consequently, patients with SLE and LN investigated in this study showed a large spectrum of AGA, predominantly the IgM isotype. Statistically significant correlations were obtained between anti-GM1 IgM and IL-6, CRP, SLEDAI, anti-dsDNA, UACR; -GD1a and IL6; -GM2 and CRP, anti-dsDNA; -GM3 and CRP, anti-dsDNA in patients with SLE. In LN patients, anti-GM1 IgM was correlated with IL-6, sIL-6R/sgp130, CRP, anti-dsDNA; -GM3, anti-dsDNA; -GD1a with CRP.

The frequency of AGA of 45.1% in SLE patients and 20.7% in LN patients showed the ability of the host to develop a specific antiganglioside immune response. Based on the present results and data in literature, causes of reduced AGA production in LN subjects could be considered the immunosuppressive effect of sialo-glycoconjugates. Our results were similar to others presented in medical literature. It was presented before that antiganglioside reactivity may act as a trigger for neurological damage in patients with systemic autoimmune disorders. A number of diseases associated with AGA have been reported: anti-GM1, anti-GD1b, anti-GQ1b IgM strongly correlated with Alzheimer’s disease, anti-GM3 and anti-GQ1b associated with multiple sclerosis, anti-GM1 with SLE, anti-GM1 or GM1-gliadin complexes with celiac disease, anti-GM2, anti-GD1a, anti GQ1b IgM isotype with HIV infection, anti-GD1b with parvovirus, anti-GM1, anti-GM2, anti-GM3, anti-GD1b, anti -GD1a with type I diabetes, anti -GM1, anti-sulfatide with Sjogren’s syndrome, rheumatoid arthritis, SLE and systemic vasculitis [17,32,34,35,36,37,38,40,41,42,43,44,52,53]. Anti-GM1 and anti-sulfatide antibodies were identified in SLE, idiopathic systemic vasculitis (VAS), Sjogren’s syndrome, and mixed cryoglobulinemia, where they did not correlate with ANA or cryocritus. A weak correlation was found between IgG isotype and SLEDAI score and anti-DNA antibodies [41]. It could not be specified the clinical impact of IgG and IgM anti-neuronal antibodies, because they have limited statistical significance with a low number of SLE activity indices. Comprehensive studies are needed to obtain detailed information about the potential role of AGA in diagnosis or prognosis of immune-mediated systemic diseases. Another study reported the presence of IgG and IgM anti-GM1 isotypes in 8% respectively 8.6%, in SLE patients. Anti-GM1-IgG and IgM antibodies identified in SLE patients could play a pathogenic role in some neuropsychiatric manifestations [53,54]. In other autoimmune conditions, a constellation of AGA has been designated [39]. It is estimated that one-third of patients with autoimmune diseases have high titers, one-third moderate titers, and one-third low titers of AGA. The majority of antibodies were polyclonal, with the predominance of the IgM isotype while anti-GM1 had the highest frequency in patients with SLE.

In conclusion, a high anti-GM1 titer could be etiologically important in certain types of neuropathies, while a low-titer antibodies could represent only B cells dysfunctional regulation [39]. In a large study, the presence of AGA antibodies (GM1, GM2, GM3, asialo-GM1, GD1a, GD1b, GD3, GT1b, GQ1b) was tested using a standard ELISA method and thin layer chromatography in a large cohort of patients with SLE over a long period of time. Positive results were found, with increased frequency, for asialo-GM1 type IgM and IgG and, with low frequency, for GM1, GM2, GM3, GD1b, GT1b, GD3 mainly IgM. Clinical and statistical studies showed no correlation between AGA and neuropsychiatric manifestations of patients with SLE [43]. Last but not least, we can appreciate that, although some patients with SLE and LN showed a broad spectrum of IgG and IgM type AGA, these antibodies are not useful markers in the diagnosis and monitoring of patients with SLE.

Some limitations of the present study should be noted. Our study followed patients with SLE and LN, for a period of three years, only with chronic immunosuppressant treatment. Further studies with a larger number of patients with different therapeutic regimens should be developed. Most of the patients included in the study had type IV nephritis. For a better evaluation of LN a larger number of patients with all types of nephritis are needed, although it would be very hard to identify these patients because they have minimum symptoms. We consider a limitation of the study having just Ig A nephropathy group as a control group. For a better understanding of LN pathogenesis, it would be necessarily the assessment of the studied markers in other nephropathies like diabetic nephritis and in sepsis. Though, further studies are needed.

In LN patients, it is very important to identify very early the patients at risk for LN development, in order to avoid invasive interventions and to establish an effective medical intervention. The present study debates an actual topic in international research, which is based on the analysis of a wide range of molecular parameters, by including several diagnostic centers, different medical specialties, with a high degree of coverage of the studied pathogenesis. This study reported that by cooperating between sialoglycolipid metabolism and IL-6 signaling, potential molecular biomarkers in LN could be identified, useful, and though, clinical and therapeutic management of patients could be improved.

## 5. Conclusions

The results of the present study showed that SLE is associated with glycoproteins and glycolipids metabolism alteration. Hypersialilation and high catabolism of glycoconjugates correlated with clinical and paraclinical data showed information about molecular mechanisms in SLE and LN. LSA/TSA ratio could be considered a molecular target for early evaluation of LN. Quantitative determination of IL-6, soluble IL-6R, sgp130 could be used in diagnosis of SLE patients, with or without renal impairment. sIL-6R:sgp 130 ratio offers information about buffering capacity of IL-6 in inflammatory response. Sgp130 quantity is higher than sIL-6R in SLE, though it is permitted both classical signaling and IL-6 mediated trans-signaling activation. Sgp130 quantity is lower than sIL-6R in LN, that restricts IL-6 mediated classical signaling. sIL-6R:sgp 130 ratio reflects differences of classical and trans-IL-6 mediated signaling in SLE and LN. Blocking IL-6 trans-signaling could prevent organs to be attacked during inflammatory events in SLE.

SLE and LN patients investigated in the study had a large AGA specter, predominantly of IgM subtype. Anti-GM1 were most frequent in SLE patients, while anti-GM1, -GM2, -GM3, -GD1a, -GD1b, -GT1b, and -GQ1b had no significant variation in our study. These results show a functional disequilibrium of immune system.

In conclusion, sialoglyco-conjugate abnormalities, high IL-6 trans-signaling and immune anti-gangliosides response profile could be considered a mechanism involved in lupus nephritis pathogenesis. By identifying and quantifying disequilibrium in sialil-conjugates metabolism during active inflammatory events, it could help toward the establishment of personalized treatments for SLE patients. Discovering how to stop inflammation and identifying control factors that react promptly would allow the body to regenerate its affected tissues.

## Figures and Tables

**Table 1:** Participants characteristics in the studied groups.

Characteristics	SLE	LN		Control	*p* Significance		
			IgA Nephropathy		SLE and LN versus Control	SLE versus LN	IgA Nephropathy versus Control
Nr Patient	62	47	32	60	>0.05	>0.05	>0.05
Women: Men ratio	2.26/1	2.35/1	2.1/1	2.33/1	>0.05	>0.05	>0.05
Age (years old)	40.3 ± 7.3	42.3 ± 6.1	39.3 ± 5.8	42.4 ± 8.0	>0.05	>0.05	>0.05
BMI (Kg/mp)	22.7 ± 1.8	23.7 ± 1.9	22.9 ± 2.4	22.3 ± 3.1	>0.05	>0.05	>0.05
Systolic Pressure (mmHg)	12.6 ± 1.4	13.3 ± 1.3	13.5 ± 1.7	11.9 ± 2.2	>0.05	>0.05	>0.05
Diastolic pressure (mmHg)	6.4 ± 0.6	7.1 ± 0.8	7.4 ± 0.9	6.9 ± 0.9	>0.05	>0.05	>0.05
Leucocytes (cells/mmc)	3700 ± 2103	4022 ± 1051	5359 ± 924	5864 ± 1078	**<0.05**	>0.05	>0.05
Hemoglobin (g/L)	10.2 ± 1.3	10.9 ± 0.9	13.1 ± 1.6	12.9 ± 1.4	**<0.05**	>0.05	>0.05
Phosphorus (mg/dL)	3.6 ± 0.9	3.5 ± 1.1	3.5 ± 0.9	3.7 ± 0.8	>0.05	>0.05	>0.05
Calcium (mg/dL)	9.16 ± 0.5	9.20 ± 0.67	9.10 ± 0.66	9.24 ± 0.59	>0.05	>0.05	>0.05
LDH(U/L)	302 ± 72	301 ± 67	307 ± 74	309 ± 61	>0.05	>0.05	>0.05
Glycemia (mg/dL)	85.7 ± 12.3	78.8 ± 14.0	3.7 ± 0.8	80.7 ± 16.4	>0.05	>0.05	>0.05
Urea (mg/dL)	36.7 ± 12.1	38.0 ± 10.7	9.24 ± 0.59	31.2 ± 7.9	>0.05	>0.05	>0.05
Creatinine (mg/dL)	1.1 ± 0.23	1.29 ± 0.23	1.09 ± 0.61	0.78 ± 0.14	**<0.05**	**<0.05**	**<0.05**
Uric acid (mg/dL)	4.4 ± 1.4	4.3 ± 1.5	4.7 ± 1.4	4.3 ± 1.3	>0.05	>0.05	>0.05
Hematuria (sw—RBC/camp)	23 ± 5	32 ± 7	10 ± 5	5 ± 5	**<0.05**	**<0.05**	**<0.05**
Leukocyturia (sw—leuc/camp)	9.1 ± 1.2	9.3 ± 1.9	2.7 ± 1.4	3.8 ± 2.9	**<0.05**	>0.05	>0.05
ASAT (U/L)	21.4 ± 13.2	19.7 ± 10.4	14.3 ± 8.3	18.2 ± 10.7	>0.05	>0.05	>0.05
ALAT (U/L)	19.1 ± 14.2	19.2 ± 7.8	15.7 ± 8.2	22.0 ± 10.5	>0.05	>0.05	>0.05
Cholesterol (mg/dL)	154.5 ± 23.5	156.2 ± 20.1	153.8 ± 22.9	146.2 ± 20.4	>0.05	>0.05	>0.05
Triglycerides (mg/dL)	91.1 ± 14.2	89.4 ± 14.2	93.2 ± 18.7	85.1 ± 16.3	>0.05	>0.05	>0.05
Albumin (g/dL)	3.23 ± 0.27	3.61 ± 0.35	4.22 ± 0.75	4.03 ± 0.46	**<0.05**	**<0.05**	>0.05
CRP(mg/dL)	2.9 ± 1.8	4.1 ± 2.7	4.6 ± 1.4	0.14 ± 0.14	**<0.05**	**<0.05**	>0.05

SLE—systemic lupus erythematosus; LN—lupus nephritis; *p*-significance level; BMI—body mass index; LDH—lactate dehydrogenase; ASAT—aspartate aminotransferase; ALAT—alanine aminotransferase; CRP—C reactive protein. We have bolded where *p* < 0.05 (significant).

**Table 2:** Disease activity parameters in the patient groups.

Parameters	SLE (62 Cases)	LN (47 Cases)	IgA Nephropathy (32 Cases)	Control (60 Cases)	*p*-Significance		
					SLE and LN versus Control	SLE versus LN	IgA Nephropathy versus Control
Disease duration (years)	7.4 ± 2.3	3.7 ± 3.1	2.4 ± 1.5	-	*-*	**<0.05**	**<0.05**
SLEDAI	5.5 ± 5.0	7.3 ± 5.1	-	-	*-*	**<0.05**	-
DNAds (UI/mL)	399.4 ± 129.7	372.7 ± 151.2	74.5 ± 23.7	79.2 ± 17.2	**<0.01**	>0.05	>0.05
Urinary b2-microglobulin (mg/L)	0.21 ± 0.09	0.36 ± 0.16	0.16 ± 0.08	0.11 ± 0.04	**<0.05**	**<0.05**	**<0.05**
UACR (mg/g creatinine)	12.89 ± 3.96	20.74 ± 3.99	11.41 ± 0.62	7.52 ± 0.43	**<0.05**	>0.05	**<0.05**
C3 (mg/dL)	66.4 ± 23.2	49.5 ± 19.8	101.7 ± 18.6	98.3 ± 12.0	**<0.01**	**<0.01**	>0.05
C4 (mg/dL)	7.0 ± 1.8	6.6 ± 1.5	18.2 ± 3.9	16.7 ± 4.3	**<0.05**	**<0.05**	>0.05
C1q (mg/dL)	4.12 ± 0.98	3.12 ± 1.3	13.7 ± 2.2	15.1 ± 3.1	**<0.05**	**<0.05**	>0.05

SLE—systemic lupus erythematosus; LN—lupus nephritis; *p*-significance level; SLEDAI—SLE Disease Activity Index; DNA ds—anti-double stranded DNA antibodies; eGFR—estimated glomerular filtration rate; UACR—urine albumin to creatinine ratio; C3—complement component 3; C4—complement component 4; C1q—complement component 1q. We have bolded where *p* < 0.05 (significant).

**Table 3:** Sialylation status in the studied groups.

Parameters	SLE (62 Cases)	LN (47 Cases)	IgA (32 cCses)	Control (60 Cases)	*p* Significance	
					p1	p2
TSA (mg/dL)	147.3 ± 28.2	244.1 ± 35.2	64.2 ± 11.0	50.04 ± 4.02	**0.002**	**AB = 0.003**
						**AD = 0.002**
						**BD = 0.002**
						**CD = 0.052**
						**BC = 0.027**
Orosomucoid (g/L)	8.11 ± 0.56	8.82 ± 0.63	1.94 ± 0.82	0.85 ± 0.17	**0.009**	AB = 0.071
						**AD = 0.004**
						**BD = 0.002**
						**CD = 0.043**
						**BC = 0.018**
LSA (mg/dL)	95.8 ± 18.4	73.5 ± 23.6	22.2 ± 4.1	18.1 ± 2.6	**0.007**	**AB = 0.041**
						**AD = 0.002**
						**BD = 0.005**
						CD = 0.215
						BC = 0.387
LSA/TSA	0.65 ± 0.15	0.30 ± 0.05	0.32 ± 0.05	0.36 ± 0.04	**0.002**	**AB = 0.007**
						**AD = 0.004**
						**BD = 0.046**
						CD = 0.071
						BC = 0.149

SLE-systemic lupus erythematosus; LN-lupus nephritis; *p*-significance level; TSA-total sialic acid; LSA-lipid bound sialic acid, *p*-statistical significance, p1-comparison of the groups, p2-pairwise comparison of the groups, A-SLE group, B- LN group, C-IgA nephropathy group, D-control group. We have bolded where *p* < 0.05 (significant).

**Table 4:** IL-6 signaling in the studied groups.

Markers	SLE (62 Cases)	LN (47 Cases)	IgA (32 Cases)	Control (C) (60 Cases)	*p* Significance	
					p1	p2
IL-6 (pg/mL)	20.9 ± 8.3	22.4 ± 10.1	14.19 ± 11.66	3.87 ± 0.56	**0.0004**	AB = 0.073
						**AD = 0.0005**
						**BD = 0.0003**
						**CD = 0.006**
						**BC = 0.014**
sIL-6R (ng/mL)	224.2 ± 57.1	248.9 ± 70.2	208.7 ± 60.9	100.1 ± 6.6	**0.007**	**AB = 0.048**
						**AD = 0.006**
						**BD = 0.004**
						**CD = 0.017**
						**BC = 0.048**
sgp 130 (ng/mL)	244.3 ± 73.5	197.4 ± 68.1	236.4 ± 51.4	314.1 ± 48.3	**0.006**	**AB = 0.034**
						**AD = 0.008**
						**BD = 0.006**
						**CD = 0.029**
						**BC = 0.011**
sIL-6R/sgp 130	0.91 ± 0.15	1.26 ± 0.12	0.88 ± 0.09	0.31 ± 0.04	**0.0008**	**AB = 0.043**
						**AD = 0.0007**
						**BD = 0.0004**
						**CD = 0.0056**
						**BC = 0.0091**

SLE-systemic lupus erythematosus; LN-lupus nephritis; *p*-significance level; IL-interleukin; sIL-6R-soluble IL-6 receptor; sgp-soluble glycoprotein. *P*-statistical significance, p1-comparison of the groups, p2-pairwise comparison of the groups, A-SLE group, B-LN group, C-IgA nephropathy group, D-control group. We have bolded where *p* < 0.05 (significant).

**Table 5:** Antiganglioside antibodies pattern in the studied groups.

AGA	Ig Class	SLE (62 Cases)	LN (47 Cases)	IgA (32 Cases)	Control (60 Cases)	*p* Significance	
						p1	p2
Anti-GM1	IgG	15.2	4.5	0.6	0.2	**0.005**	**AB = 0.004** **AD = 0.025** **BD = 0.042** CD = 0.056 **BC = 0.047**
	IgM	31.3	17.3	8.3	1.1	**0.006**	**AB = 0.007** **AD = 0.026** **BD = 0.033** **CD = 0.029** **BC = 0.012**
Anti-GM2	IgG	4.3	0.2	0.05	0.05	**0.049**	**AB = 0.035** **AD = 0.023** **BD = 0.042** CD = 0.872 BC = 0.146
	IgM	26.1	7.8	0.02	0.05	**0.007**	**AB = 0.005** **AD = 0.012** **BD = 0.017** CD = 0.341 **BC = 0.045**
Anti-GM3	IgG	8.3	0.3	0.4	0.05	**0.037**	**AB = 0.021** **AD = 0.036** **BD = 0.047** CD = 0.062 BC = 0.057
	IgM	24,3	11.6	0.10	0.2	**0.006**	**AB = 0.004** **AD = 0.025** **BD = 0.037** CD = 0.239 **BC = 0.042**
Anti-GD1a	IgG	0.2	0.05	0.07	0.05	0.461	AB = 0.072 AD = 0.187 BD = 0.392 CD = 0.357 BC = 0.738
	IgM	7.3	3.4	0.05	0.05	**0.007**	**AB = 0.007** **AD = 0.029** **BD = 0.036** CD = 0.459 **BC = 0.043**
Anti-GD1b	IgG	0.1	0.05	0.10	0.05	0.592	AB = 0.284 AD = 0.392 BD = 0.891 CD = 0.314 BC = 0.462
	IgM	4.1	7.2	1.0	0.05	**0.084**	**AB = 0.067** **AD = 0.032** **BD = 0.043** CD = 0.266 **BC = 0.022**
Anti-GT1b	IgG	0.3	0.2	0.30	0.1	0.492	AB = 0.577 AD = 0.162 BD = 0.787 CD = 0.596 BC = 0.347
	IgM	5.7	9.1	0.04	0.05	**0.041**	**AB = 0.011** **AD = 0.032** **BD = 0.017** CD = 0.731 **BC = 0.017**
Anti-GQ1b	IgG	0.05	0.2	1.2	0.05	0.183	AB = 0.357 AD = 0.971 BD = 0.274 CD = 0.149 BC = 0.072
	IgM	8.2	0.1	0.1	0.1	**0.049**	**AB = 0.035** **AD = 0.038** BD = 0.952 CD = 0.872 BC = 0.964

SLE-systemic lupus erythematosus; LN-lupus nephritis; *p*-significance level; AGA-anti-ganglioside antibodies; Ig- immunoglobulin; GM1 = Gal-3GalNAc-4(Neu5Ac-3)Gal-4GlcCer; GM2 = GalNAc-4(Neu5Ac-3)Gal-4GlcCer; GM3 = Neu5Ac-3Gal-4GlcCer; GD1a = Neu5Ac-3Gal-3GalNAc-4(Neu5Ac-3)Gal-4GlcCer; GD1b = Gal-3GalNAc-4(Neu5Ac-8Neu5Ac-3)Gal-4GlcCer; GT1b = Neu5Ac-3Gal-3GalNAc-4(Neu5Ac-8Neu5Ac-3)Gal-4GlcCe; GQ1b = Neu5Ac-8Neu5Ac-3Gal-3GalNAc-4(Neu5Ac-8Neu5Ac-3)Gal-4GlcCer; Glc = glucose; Gal= galactose; GalNAc = N-acetyl-galactosamine. *p*-statistical significance, p1-comparison of the groups, p2-pairwise comparison of the groups, A-SLE group, B-LN group, C-IgA nephropathy group, D-control group. We have bolded where *p* < 0.05 (significant).

**Table 6:** Correlation analysis between glycoconjugates/antigangliosides and inflammatory markers.

Parameters	IL-6	sIL-6R	sgp130	sIL-6R/sgp130	CRP
SLE (62 cases)					
TSA	*r* = 0.88 (x)	NS	NS	NS	*r* = 0.37 (xx)
Orosomucoid	*r* = 0.66 (x)	*r* = 0.28 (xx)	NS	NS	*r* = 0.82 (x)
LSA	NS	NS	NS	NS	*r* = 0.10 (xx)
Ig M-anti-GM1	*r* = 0.43 (x)	NS	NS	NS	*r* = 0.19 (xx)
IgM-anti-GM2	NS	NS	NS	NS	*r* = −0.26 (xx)
IgM -anti-GM3	NS	NS	NS	NS	*r* = −0.27 (xx)
IgM-anti-GD1a	*r* = 0.19 (xx)	NS	NS	NS	NS
IgM-anti-GD1b	NS	NS	NS	NS	NS
IgM-anti-GT1b	NS	NS	NS	NS	NS
IgM-anti-GQ1b	NS	NS	NS	NS	NS
sIL6-R	NS	-	NS	NS	NS
sgp130	NS	NS	-	NS	NS
CRP	*r* = 0.68 (x)	NS	NS	NS	-
LN (47 cases)					
TSA	*r* = 0.25 (xx)	NS	NS	NS	*r* = −0.43 (x)
Orosomucoid	*r* = 0.77 (x)	*r* = −0.34 (xx)	NS	NS	*r* = 0.53 (xx)
LSA	*r* = −0.16 (xx)	NS	NS	*r* = −0.31 (xx)	NS
Ig M-anti-GM1	*r* = 0.26 (xx)	NS	NS	*r* = 0.47 (x)	*r* = 0.11 (xxx)
IgM-anti-GM2	NS	NS	NS	NS	NS
IgM-anti-GM3	NS	NS	NS	NS	NS
IgM-anti-GD1a	NS	NS	NS	NS	*r* = 0.41 (x)
IgM-anti-GD1b	NS	NS	NS	NS	NS
IgM-anti-GT1b	NS	NS	NS	NS	NS
IgM-anti-GQ1b	NS	NS	NS	NS	NS
sIL-6R	*r* = 0.42 (xx)	-	NS	NS	*r* = 0.56 (xx)
sgp130	NS	NS	-	NS	NS
CRP	*r* = 0.87 (x)	*r* = 0.56 (xx)	NS	NS	-

SLE—systemic lupus erythematosus; LN—lupus nephritis; *p*-significance level; NS—insignificant; *r*—correlation coefficient; IL—interleukin; sIL-6R-soluble IL-6 receptor; sgp—soluble glycoprotein; CRP—protein R-reactive; Ig—immunoglobulin; TSA—total sialic acid; LSA—lipid sialic acid; GM1—Gal-3GalNAc-4(Neu5Ac-3)Gal-4GlcCer; GM2—GalNAc-4(Neu5Ac-3)Gal-4GlcCer; GM3—Neu5Ac-3Gal-4GlcCer; GD1a—Neu5Ac-3Gal-3GalNAc-4(Neu5Ac-3)Gal-4GlcCer; GD1b—Gal-3GalNAc-4(Neu5Ac-8Neu5Ac-3)Gal-4GlcCer; GT1b—Neu5Ac-3Gal-3GalNAc-4(Neu5Ac-8Neu5Ac-3)Gal-4GlcCe; GQ1b—Neu5Ac-8Neu5Ac-3Gal-3GalNAc-4(Neu5Ac-8Neu5Ac-3)Gal-4GlcCer; Glc—glucose; Gal—galactose; GalNAc—N-acetyl-galactosamine; (x)—*p* < 0.01; (xx)—*p* < 0.05; (xxx)—*p* = 0.05 The correlations were assessed by Pearson’s coefficient, after assessment of data normality by the Kolmogorov–Smirnov test.

**Table 7:** Correlation analysis between glycoconjugates/antigangliosides and disease activity markers.

Parameters	SLEDAI	Anti-DNAds	C3	UACR	B2microglobulin	C1q
SLE (62 cases)						
TSA	*r* = 0.35 (xx)	*r* = 0.42 (x)	*r* = −0.54 (x)	*r* = 0.11 (xx)	*r* = 0.21 (xx)	*r* = −0.34 (xx)
Orosomucoid	*r* = 0.53 (x)	*r* = 0.38 (xx)	NS	*r* = 0.21 (xx)	NS	NS
LSA	*r* = 0.38 (xx)	*r* = 0.39 (xxx)	NS	*r* = 0.16 (xx)	NS	*r* = −0.17 (xx)
IgM-anti-GM1	*r* = 0.28 (xx)	*r* = 0.48 (xx)	*r* = −0.33 (xxx)	NS	NS	NS
IgM-anti-GM2	NS	*r* = 0.12 (xxx)	NS	NS	NS	NS
IgM-anti-GM3	NS	*r* = 0.10 (xxx)	NS	NS	NS	NS
IgM-anti-GD1a	NS	NS	NS	NS	NS	NS
IgM-anti-GD1b	NS	NS	NS	NS	NS	NS
IgM-anti-GT1b	NS	NS	NS	NS	NS	NS
IgM-anti-GQ1b	NS	NS	NS	NS	NS	NS
LN (47 cases)						
TSA	*r* = 0.37 (xx)	*r* = 0.72 (x)	*r* = −0.39 (xx)	*r* = 0.57 (x)	*r* = 0.42 (xx)	*r* = −0.51 (x)
Orosomucoid	*r* = 0.76 (x)	*r* = 0.37 (xx)	NS	*r* = 0.12 (xx)	NS	NS
LSA	*r* = 0.51 (x)	NS	NS	*r* = 0.64 (xx)	NS	NS
IgM-anti-GM1	NS	*r* = 0.65 (xx)	NS	NS	NS	NS
IgM-anti-GM2	NS	NS	NS	NS	NS	NS
IgM-anti-GM3	NS	*r* = 0.08 (xxx)	NS	NS	NS	NS
IgM-anti-GD1a	NS	NS	NS	NS	NS	NS
IgM-anti-GD1b	NS	NS	NS	NS	NS	NS
IgM-anti-GT1b	NS	NS	NS	NS	NS	NS
IgM-anti-GQ1b	NS	NS	NS	NS	NS	NS

SLE—systemic lupus erythematosus; LN—lupus nephritis; *p*—significance level; *r*—correlation coefficient; NS—insignificant; SLEDAI—SLE Disease Activity Index; ADNds—anti-double stranded DNA antibodies; UACR—urine albumin to creatinine ratio; C3—complement component 3; C1q—complement component 1q; Ig—immunoglobulin; TSA—total sialic acid; LSA—lipid bound sialic acid; GM1—Gal-3GalNAc-4(Neu5Ac-3)Gal-4GlcCer; GM2—GalNAc-4(Neu5Ac-3)Gal-4GlcCer; GM3—Neu5Ac-3Gal-4GlcCer; GD1a—Neu5Ac-3Gal-3GalNAc-4(Neu5Ac-3)Gal-4GlcCer; GD1b—Gal-3GalNAc-4(Neu5Ac-8Neu5Ac-3)Gal-4GlcCer; GT1b—Neu5Ac-3Gal-3GalNAc-4(Neu5Ac-8Neu5Ac-3)Gal-4GlcCe; GQ1b—Neu5Ac-8Neu5Ac-3Gal-3GalNAc-4(Neu5Ac-8Neu5Ac-3)Gal-4GlcCer; Glc—glucose; Gal—galactose; GalNAc—N-acetyl-galactosamine; (x)—*p* < 0.01; (xx)—*p* < 0.05; (xxx)—*p* = 0.05. The correlations were assessed by Pearson’s coefficient, after assessment of data normality by the Kolmogorov–Smirnov test.

## Data Availability

All data is presented in the paper.
